# Fertility Desires among Women Living with HIV

**DOI:** 10.1371/journal.pone.0160190

**Published:** 2016-09-09

**Authors:** Deborah Lynne Jones, Ryan Cook, JoNell Efantis Potter, Talya Miron-Shatz, Nahida Chakhtoura, Andrew Spence, Margaret M. Byrne

**Affiliations:** 1 Psychiatry and Behavioral Sciences, University of Miami Miller School of Medicine, Miami, Florida, United States of America; 2 Obstetrics and Gynecology, University of Miami Miller School of Medicine, Miami, Florida, United States of America; 3 Ono Academic College, Kiryat Ono, Israel; 4 Center for Medicine in the Public Interest, New York, New York, United States of America; 5 Epidemiology and Public Health, University of Miami Miller School of Medicine, Miami, Florida, United States of America; University of Pittsburgh, UNITED STATES

## Abstract

**Objective:**

Rates of pregnancy among women living with HIV (WLHIV) have increased with the availability of effective HIV treatment. Planning for pregnancy and childbirth is an increasingly important element of HIV care. Though rates of unintended pregnancies are high among women in general, among couples affected by HIV, significant planning and reproductive decisions must be considered to prevent negative health consequences for WLHIV and their neonates. To gain insight into this reproductive decision-making process among WLHIV, this study explored women’s knowledge, attitudes and practices regarding fertility planning, reproductive desires, and safer conception practices. It was hypothesized that pregnancy desires would be influenced by partners, families, the potential risk of HIV transmission to infants, and physicians’ recommendations.

**Methods:**

WLHIV of childbearing age were recruited from urban South Florida, and completed an assessment of demographics (*N* = 49), fertility desires and a conjoint survey of factors associated with reproductive decision-making.

**Results:**

Using conjoint analysis, we found that different decision paths exist for different types of women: Younger women and those with less education desired children if their partners wanted children; reproductive desires among those with less education, and with less HIV pregnancy-related knowledge, displayed a trend toward additional emphasis on their family’s desires. Conversely, older women and those with more education appeared to place more importance on physician endorsement in their plans for childbearing.

**Conclusions:**

Results of this study highlight the importance of ongoing preconception counselling for all women of reproductive age during routine HIV care. Counselling should be tailored to patient characteristics, and physicians should consider inclusion of families and/or partners in the process.

## Introduction

Rates of pregnancy among women living with HIV (WLHIV) have increased with the availability of HIV treatment, and planning for pregnancy is an increasingly important element of HIV care [1]. Antiretroviral therapy (ART) has greatly reduced perinatal transmission of HIV, and increasing numbers of WLHIV are conceiving [2–7]. Rates of unintended pregnancies among WLHIV seem to run parallel to those of women without HIV [8, 9]. Unplanned pregnancies may carry a high risk for negative health consequences for couples affected by HIV and their neonates, including an increased risk for HIV acquisition for partners and neonates as well as maternal and neonatal mortality. Among pregnant WLHIV surveyed in the United States [9], only 36% of couples had discussed and agreed on their last pregnancy. Many couples had no desire to become pregnant (50%), and 33% had not sought medical advice to prepare for pregnancy, taken prenatal vitamins or decreased substance use. Most reported using inconsistent or no contraception in the month they became pregnant (68%). In fact, women with a history of unplanned pregnancy have reported being less likely to seek contraception [10], and not surprisingly, many reported becoming pregnant without clinical consultation (49%) [11]. It appears that some women may neither plan nor prevent pregnancy [12], and some women may hold an intention to conceive that may be actualized at any time due to inconsistent or non-existent contraceptive use.

Preconception counselling for WLHIV, i.e., assessment of fertility intentions and presentation of health recommendations for conception, may reduce the risk of foetal loss, preterm delivery, low birth weight, birth defects, and vertical and horizontal transmission of HIV [13]. Preconception counselling targets pregnancy planning and health recommendations, but rarely addresses safer conception methods, e.g., methods to reduce the risk of horizontal or vertical HIV transmission [11]. Despite the need for preconception counselling, which may address mutual HIV serostatus disclosure, use of Pre-Exposure Prophylaxis (PrEP) or ART, timed intercourse, women may avoid discussing fertility desires with healthcare providers. WLHIV may also anticipate healthcare providers to have negative attitudes towards childbearing [1, 14, 15]. While women may be less likely to have an unplanned pregnancy after discussing pregnancy intentions with their healthcare provider [9], communication in the clinical setting about fertility intentions may be brief or neglected [3], and visits may focus on contraception rather than conception.

Interpersonal and social factors, such as gender expectations for women to bear children, and the desires of family members and partners for the woman to have a child, influence a woman’s own desire to conceive [16–18]. In fact, perceived partner desire may play a more influential role than healthcare provider guidance [2–6]. These multiple influences on reproductive decision-making and potential gaps in knowledge highlight the need to explore how and with whom women reach reproductive decisions, and what can be done to improve these paths. As such, this pilot study was designed to examine the drivers of reproductive decision-making among WLHIV that underlie a woman’s intention to conceive, and identify targets for preconception counselling. It was hypothesized that outside influences on women’s desires for pregnancy could conflict and compete with recommendations from healthcare providers. By assessing competing alternatives that may be evaluated by WLHIV during the reproductive decision-making process, results from this study provide novel insight to inform preconception counselling strategies and reproductive healthcare provision for WLHIV, thereby enhancing health outcomes for women, partners and neonates.

## Methods

### Participants and Procedures

University of Miami Miller School of Medicine institutional review board approval was obtained prior to study onset. All participants provided written informed consent prior to enrolment. Participants (*N* = 49) were recruited from community health centres, and outpatient clinics at public and private hospitals by word-of-mouth in urban South Florida. Eligible participants were WLHIV aged 18–45, sexually active within the past six months, non-pregnant, fluent in English, and capable of conception (no history of tubal ligation or hysterectomy). Of 97 screened for eligibility, 55 were eligible to participate, and 49 enrolled. Reasons for ineligibility were age (*n* = 16), HIV seronegative (*n* = 11), history of tubal ligation or hysterectomy (*n* = 9), and lack of sexual activity in the past 6 months (*n* = 6). Participants were compensated $30 for time and travel to the site; all interviews were held immediately following provision of informed consent in offices adjacent to the public hospital. Study personnel conducted assessments in a private office using an audio computer assisted self-interview (ACASI) system to accommodate all levels of literacy. The survey required 30 to 45 minutes to complete, and was administered in English. Study personnel guided the participant in learning to use the ACASI and were available in the next office at all times.

### Measures

Participant questionnaires were developed by a team of healthcare providers in psychology, obstetrics/gynaecology, infectious diseases and epidemiology, and supplemented by informal groups and discussions with hospital healthcare providers, scientists and patients.

Demographic information, current health status, communication with others regarding conception and HIV status (disclosure), reproductive knowledge, and conception attitudes, practices and desires were assessed. In addition, two questions addressed knowledge of the potential for ART to lower the risk of HIV transmission to partners and infants, knowledge was dichotomized into “high” (answered both questions correctly) and “low” (answered at least one question incorrectly) levels. The DelibeRATE scale [19], a 9-item measure using a Likert scale ranging from “1, strongly disagree” to “7, strongly agree”, was used to assess the level of thought participants had put into the consideration of safe conception practices, such as whether the women felt they had the information needed to make a decision of which method to use.

#### Conjoint survey

A conjoint survey was used to quantify the relative importance of the attributes involved in a decision-making process. Conjoint surveys more closely represent real-life decision-making, in which attributes of a situation exist as part of a group of factors and “trade-offs” must be made by the individual making the decision [20–24], and are increasingly being utilized in health research [25–28]. Five attributes were selected by the team as potential determinants of reproductive decision-making: the cost of having children, partner opinion, family opinion, the potential of HIV transmission to partner, and healthcare provider opinion. The risk of HIV transmission to infants was not included in the survey due to its high degree of importance to all of the women interviewed; there was concern that this attribute would limit variability in participant responses, hindering statistical analysis. “Levels,” positive (e.g., participant will gain income from having a baby), negative (e.g., the partner might get HIV), and neutral (e.g., having a baby was not discussed by healthcare provider) were assigned to each attribute. Given the combination of five attributes and 2–3 levels assigned to each attribute, 108 unique profiles of combinations of attributes were possible, representing a scenario about a woman living with HIV and pregnancy/childbirth decision making that reflected the attribute levels defined in the profile. Of the 108 profiles, a subset of 12 were selected (see Table 1) to achieve an optimal experimental design for a partial-profile choice model, in which in each choice set, only a subset of the attributes vary and the rest remain constant [29].

**Table 1:** Conjoint survey.

How much you would want to have a baby if …?	
**0.** Having a baby would COST you money. Your partner DOES NOT WANT a baby. Your family WANTS you to have a baby. Having a baby MIGHT INFECT your partner with HIV. Your doctor DID NOT TALK with you about having a baby.	Please choose from 1 (*Would not want a baby*) to 10 (*Would really want a baby*).
**1.** Having a baby would COST you money. Your partner WANTS a baby. Your family WANTS you to have a baby. Having a baby MIGHT INFECT your partner with HIV. Your doctor APPROVES of you having a baby.	Please choose from 1 (*Would not want a baby*) to 10 (*Would really want a baby*).
**2.** Having a baby would COST you money. Your partner WANTS a baby. Your family DOES NOT CARE if you have a baby. Having a baby would NOT INFECT your partner with HIV. Your doctor APPROVES of you having a baby.	Please choose from 1 (*Would not want a baby*) to 10 (*Would really want a baby*).
**3.** Having a baby would COST you money. Your partner DOES NOT WANT a baby. Your family WANTS you to have a baby. Having a baby MIGHT INFECT your partner with HIV. Your doctor DID NOT TALK with you about having a baby.	Please choose from 1 (*Would not want a baby*) to 10 (*Would really want a baby*).
**4.** You would GET MONEY for having a baby. Your partner WANTS a baby. Your family WANTS you to have a baby. Having a baby would NOT INFECT your partner with HIV. Your doctor DID NOT TALK with you about having a baby.	Please choose from 1 (*Would not want a baby*) to 10 (*Would really want a baby*).
**5.** You would GET MONEY for having a baby. Your partner DOES NOT CARE about having a baby. Your family WANTS you to have a baby. Having a baby MIGHT INFECT your partner with HIV. Your doctor APPROVES of you having a baby.	Please choose from 1 (*Would not want a baby*) to 10 (*Would really want a baby*).
**6.** You would GET MONEY for having a baby. Your partner DOES NOT WANT a baby. Your family DOES NOT WANT you to have a baby. Having a baby would NOT INFECT your partner with HIV. Your doctor DISAPPROVES of you having a baby.	Please choose from 1 (*Would not want a baby*) to 10 (*Would really want a baby*).
**7.** You would GET MONEY for having a baby. Your partner DOES NOT CARE about having a baby. Your family WANTS you to have a baby. Having a baby would NOT INFECT your partner with HIV. Your doctor APPROVES of you having a baby.	Please choose from 1 (*Would not want a baby*) to 10 (*Would really want a baby*).
**8.** Having a baby would COST you money. Your partner WANTS a baby. Your family WANTS you to have a baby. Having a baby would NOT INFECT your partner with HIV. Your doctor DISAPPROVES of you having a baby.	Please choose from 1 (*Would not want a baby*) to 10 (*Would really want a baby*).
**9.** Having a baby would COST you money. Your partner DOES NOT WANT a baby. Your family WANTS you to have a baby. Having a baby would NOT INFECT your partner with HIV. Your doctor DID NOT TALK with you about having a baby.	Please choose from 1 (*Would not want a baby*) to 10 (*Would really want a baby*).
**10.** Having a baby would COST you money. Your partner DOES NOT CARE about having a baby. Your family DOES NOT CARE if you have a baby. Having a baby MIGHT INFECT your partner with HIV. Your doctor DISAPPROVES of you having a baby.	Please choose from 1 (*Would not want a baby*) to 10 (*Would really want a baby*).
**11.** You would GET MONEY for having a baby. Your partner WANTS to have a baby. Your family DOES NOT WANT you to have a baby. Having a baby MIGHT INFECT your partner with HIV. Your doctor DID NOT TALK with you about having a baby.	Please choose from 1 (*Would not want a baby*) to 10 (*Would really want a baby*).
**12.** You would GET MONEY for having a baby. Your partner WANTS to have a baby. Your family WANTS you to have a baby. Having a baby MIGHT INFECT your partner with HIV. Your doctor DISAPPROVES of you having a baby.	Please choose from 1 (*Would not want a baby*) to 10 (*Would really want a baby*).

For each scenario (see Table 1), participants were asked to rate how much they would want to have a baby on a scale of (1), would not want to have a baby, to (10) would really want to have a baby. The scenarios were introduced with a sample hypothetical situation that was the result of a combination of all negative attribute levels, “The following questions address how much you might want to have a baby. The questions include five things that might affect your decision. Listen carefully because the topics in each question are different. The following things are all negative. How much would you want to have a baby if… Having a baby would COST you money, Your partner DOES NOT WANT a baby, Your family DOES WANT you to have a baby, Having a baby MIGHT INFECT your partner with HIV, Your doctor DISAPPROVES of you having a baby”. Pictorial depictions of each scenario were provided to enhance comprehension (e.g., a “negative” partner response was depicted by a picture of an unhappy young adult man). The participant was then asked to rate another scenario, “Now, some of these things are positive, so your feelings may be different”.

### Analysis of the Conjoint Data

Conjoint data is analysed using many different methods, depending on the type of conjoint survey utilized [30]. As this survey utilized a metric rating scale, the expected preference for any given profile was modelled as the sum of a constant plus the parameter estimates associated with the levels of the attributes appearing in that profile, which were estimated using ordinary least-squares regression techniques. Scenario ratings were the outcome values in the regression, and the predictors were the attributes appearing in the scenarios. The parameter estimates corresponding to each level of each attribute (called “part-worth utilities”) were estimated using ordinary least squares multiple regression on an individual level (i.e., a separate analysis per subject).

Using part-worth utilities, “importance scores” were computed for each subject by calculating the absolute value of the difference between the largest part-worth utility and the smallest part-worth utility associated with the levels of each attribute. For a two level attribute (such as income in this study), this was simply the range of the part-worth utilities estimated for each of the two levels. The utility range was then expressed as a percentage of the sum total of all utility ranges of all attributes, which resulted in an importance score for each attribute, describing the importance of that attribute relative to the others [20, 24, 31]. Importance scores were then averaged across study participants and summarized using descriptive statistics to characterize the influence of the attributes on participant desire to become pregnant. Additionally, importance scores were tested for association with demographic, pregnancy, and HIV-related characteristics using Wilcoxon rank-sum tests and Kendall Tau correlation coefficients.

The validity of the conjoint data gathered was tested on a subsample of *n* = 20 women using “holdout” profiles [21, 32]. Holdout profiles measure how well utilities resulting from a conjoint model predict actual participant ratings. In this study, results from holdout profiles indicated that there was not a significant difference between model predictions and actual ratings (*b* = -.381, *se* = .206, *p =* .080), indicating that the validity of the conjoint data was adequate. All statistical analyses were completed using SAS v.9.3.

## Results

### Participant Characteristics

Participants (*N* = 49) were primarily African-American (70%) and ranged from 18–45 years of age (*M* = 36; *SD* = 8). Participants had 11 ± 2 years of education and the majority were unemployed (90%). Most were not married but in a relationship (60%) and had children (73%). The majority of participants desired additional children (82%), and of those, nearly half were actively trying to become pregnant (48%). Most women reported that they had discussed becoming pregnant with their healthcare provider (67%). Participants reported having lived with HIV for an average of 13 ± 6 years and nearly all were taking ART (94%); however, only about a third self-reported an undetectable viral load (37%). Of those with a current partner, most knew their partner’s serostatus (87%). Of those aware of partner status, just under half indicated that their partner was also HIV-infected (46%). Detailed participant characteristics are presented in Table 2.

**Table 2:** Demographic, pregnancy and HIV-related characteristics of N = 49 urban multiethnic women with HIV.

Characteristic	Range, Mean(*SD*) n(%)
Age	18–45, 36(8)
Years of education	5–16, 11(2)
**Ethnicity**	
Hispanic	7(14%)
African-American	34(70%)
Non-Hispanic White	2(4%)
Other	6(12%)
**Employment status**	
Employed full time/part time	5(10%)
Unemployed/Volunteering	44(90%)
**Personal monthly income**	
Less than $300	14(29%)
More than $300	35(71%)
**Disability/welfare status**	
Receiving disability/welfare	31(63%)
Not receiving disability/welfare	18(37%)
**Marital/relationship status**	
Single, no current partner	7(14%)
Not married, but have a partner	29(60%)
Married	9(18%)
Other- separated, widowed, etc.	4(8%)
**Children**	
Have children	36(73%)
Do not have children	13(27%)
**Want (more) children**	
Yes	40(82%)
No	9(18%)
**Actively trying to become pregnant** (*n =* 40 who want more children)	
Yes	19(48%)
No	21(52%)
**Discussed pregnancy with current partner** (*n =* 38 with a current partner)	
Yes	31(82%)
No	7(18%)
**Discussed pregnancy with family**	
Yes	15(31%)
No	34(69%)
**Discussed pregnancy with a healthcare provider**	
Yes	33(67%)
No	16(33%)
**Number of years since HIV diagnosis** (*n =* 44 provided a date)	0–23, 13(6)
**Had a viral load test in past 6 months**	
Yes	41(85%)
No	8(16%)
**Undetectable viral load** (self-reported)	
Yes	18(37%)
No	31(63%)
**On ART**	
Yes	46(94%)
No	3(6%)
**Disclosed HIV status to partner** (*n* = 38 with current partner)	
Yes	37(97%)
No	1(3%)
**Knowledge of current partner’s HIV serostatus**	
Yes	33(87%)
No	5(13%)
**Partner is HIV seropositive**	
Yes	15(46%)
No	18(55%)

### Family Planning and Reproductive Decision-Making

A small number of participants (*n* = 6) provided the same rating across all profiles. As these participants had no outcome variability and variability in response ratings was needed to calculate part-worth utilities using ordinary least-squares regression, they were excluded from conjoint analyses, resulting in *n* = 43 (see Table 3). Positive mean utilities on factor levels indicate that those levels were associated with increased desire for pregnancy, on average, with the converse true for negative mean utilities. Examination of the confidence intervals on the utilities demonstrated that positive partner’s opinions, provider’s opinions, and no risk of HIV transmission were consistently associated with increased desire for children (i.e., the lower bounds on the confidence intervals were positive). Similarly, negative opinions on those factors were consistently associated with decreased desire (i.e., the upper bounds were negative). Other attributes, such as positive family opinion, had confidence intervals including zero. This indicates that the impact of these attributes were inconsistent, resulting in an increased desire for children in some and a decreased desire in others.

**Table 3:** Utilities associated with each attribute of participants’ desire to become pregnant (n = 43 with variability in profile ratings).

Attribute Level	Utility range	Mean utility (*SD*)	95% CI (Mean utility)
**Family**			
Family approves	-2.94	.01(.55)	(-.16, .18)
Family doesn’t care	-6.21	.11(1.23)	(-.27, .49)
Family disapproves	-7.13	-.12(1.27)	(-.51, .27)
**Provider**			
Provider approves	-5.1	.52(1.05)	(.20, .84)
Provider hasn’t discussed	-4.4	-.11(.82)	(-.37, .13)
Provider disapproves	-4.4	-.40(.82)	(-.65, -.15)
**Partner**			
Partner approves	-3.22	.42(.64)	(.22, .62)
Partner doesn’t care	-3.88	-.04(.79)	(-.28, .21)
Partner disapproves	-4.78	-.38(1.03)	(-.70, -.06)
**HIV**			
No chance to infect partner	-4.09	.56(.78)	(.32, .80)
Chance to infect partner	-4.09	-.56(.78)	(-.32, -.80)
**Income**			
Gain income	-6.12	-.06(.89)	(-.33, .22)
Lose income	-6.12	.06(.89)	(-.22, .33)

Results demonstrated that the family’s opinion (mean importance = 24.6%), provider’s opinion (23.7%), and partner’s desire for a child (23.5%) were the most influential factors in the decision-making process, relative to risk of HIV transmission (15.6%) and potential to increase/decrease income (12.5%). Additionally, since the importance of factors was relative other factors within each person, rankings were created for each participant. Among one third of participants (*n* = 14), partner desire was the most influential factor. Equal numbers of participants weighed their provider’s (26%) and family’s opinion (26%) most significantly (see Table 4).

**Table 4:** Relative importance of factors influencing participants’ desire to become pregnant (*n =* 43 with variability in profile ratings).

Factor	Importance range	Mean importance (*SD*)	95% CI (Mean importance)	n(%) ranking #1 importance
Family opinion	5% - 51%	24.6(12.6)	20.8–28.5	11(26%)
Health care provider opinion	4% - 73%	23.7(13.4)	19.6–28.9	11(26%)
Partner desire	3% - 54%	23.5(11.9)	19.8–27.2	14(33%)
Risk of HIV transmission to partner	0% - 83%	15.6(14.8)	11.1–20.2	4(9%)
Potential to increase/decrease income	0% - 44%	12.5(10.1)	9.4–15.6	3(7%)

Importance scores were compared between groups of women sharing selected common demographic, pregnancy, and HIV-related characteristics (see Table 5). Age was negatively correlated with the importance of the partner’s opinion (*p =* .050), and women with higher levels of education placed more emphasis on provider opinion (*p =* .045). Women who were actively trying to become pregnant placed more importance on the potential to gain or lose income (*p =* .001), compared to those who desired additional children but were not actively trying to become pregnant.

**Table 5:** Mean importance scores by demographic, pregnancy, and HIV-related characteristics (n = 43 with variability in profile ratings).

	Income *M*(*SD*)	Partner	Family	HIV transmission	Provider
**Education level**					
Finished high school (*n =* 19)	10.8(8.9)	21.5(12.5)	21.6(14.3)^	17.7(18.7)	28.3(14.4)*
Did not finish high school (*n =* 24)	13.9(10.9)	25.1(11.4)	26.9(11.0)	13.9(11.1)	20.1(11.7)
**Relationship status**					
Have current partner (*n =* 33)	12.7(9.7)	24.2(11.3)	23.8(11.4)	15.1(11.3)	24.2(14.4)
No current partner (*n =* 10)	12.0(11.7)	21.1(14.1)	27.3(16.3)	17.3(23.8)	22.2(10.1)
**Actively trying to become pregnant** (*n =* 35 who want children)					
Yes (*n =* 14)	18.9(12.2)**	21.3(11.1)	27.9(9.8)	10.9(6.5)	21.0(10.3)
No (*n =* 21)	8.2(7.2)	24.4(13.5)	22.3(15.0)	19.7(19.2)	25.4(16.0)
**Discussed pregnancy with a healthcare provider**					
Yes (*n =* 29)	11.9(11.0)	23.1(13.6)	24.8(14.3)	17.3(17.5)	22.9(15.0)
No (*n =* 14)	13.7(7.9)	24.4(7.7)	24.2(8.4)	12.2(5.3)	25.4(9.7)
**Undetectable viral load (self-reported)**					
Yes (*n =* 17)	10.7(10.3)	22.2(10.2)	27.2(14.0)	14.3(10.8)	22.4(10.3)
No (*n =* 26)	13.7(9.9)	24.3(13.0)	23.0(11.5)	17.6(19.9)	24.6(15.3)
**Knowledge level**					
High (*n =* 24)	11.1(8.1)	23.9(13.3)	21.6(12.1)^	17.7(18.7)	25.6(15.0)
Low (*n =* 19)	14.2(12.1)	23.0(10.2)	28.4(12.5)	13.0(7.0)	21.3(11.1)
Correlations between importance scores and age and number of years since HIV diagnosis					
	Tau, *p*	Tau, *p*	Tau, *p*	Tau, *p*	Tau, *p*
Age	0.08, .48	**-.21, .05**	.16, .12	.06, .60	-.07, .51
Time since diagnosis (n = 40)	0.07, .55	-.11, .32	-.13, .25	.12, .29	-.03, .82

Note.

^*p* < .10

*p < .05

**p < .01

***p < .001 by Wilcoxon test.

### Knowledge and Readiness for Decision-Making

Many women reported they felt they had enough information about the issues surrounding HIV and pregnancy to reach a decision to conceive (mean score 47.8 ± 15.6, range 9 to 63, maximum possible 63). Lastly, DelibeRATE scores were similar between those desiring more children, those actively trying to become pregnant, and those with higher or lower levels of knowledge surrounding HIV transmission during pregnancy.

## Discussion

This study examined factors and underlying intentions to conceive among WLHIV, and identified desires to conceive, a lack of safer conception practices, and potential targets for preconception counselling. The effects of partners and healthcare providers on decision-making were consistent with theory (e.g., a positive partner’s opinion was associated with higher desire for a child) and across participants, indicating that these factors were influential to nearly all participants. Thus, results suggest that partners and providers significantly influence women’s decision to become pregnant, as found in previous research [17]. Despite the large importance score associated with family opinion, its influence was inconsistent, with women associating positive family opinions with both higher and lower desires for children. This may reflect the complex family dynamics experienced by WLHIV, as some report estrangement from families. The majority of women did not appear to be influenced by transmission risk to partners, or by financial stressors associated with having children. However, the importance placed on transmission risk to partners may have been minimized in this sample given that nearly half of the women reported that their partner was HIV-infected. Nevertheless, preconception counselling, which typically addresses conception from the patient’s perspective, should highlight the potentially negative consequences for sexual partners.

Though many women felt they had the information on conception and HIV they needed to make decision regarding conception, in fact, the women in this sample who most desired a child had less than adequate information regarding the complicated issues surrounding healthy pregnancy for WLHIV. This underscores both the importance of childbearing among the women in this sample and the challenges for preconception counselling among WLHIV. Women may make up their minds to have a child and become actively engaged in the process, such that the desire to have a child quickly outweighs information presented by providers. Preconception counselling has been recommended for all WLHIV of childbearing age [13, 33], and results of this study suggest that healthcare providers, e.g., primary care providers, infectious disease physicians, obstetricians and gynaecologists, should open the preconception dialogue with patients frequently and early to address gaps in knowledge [1, 34, 35]. By making preconception and counselling packages available during clinical consultations [36–37], women may be able to share the knowledge and information with their family and partners, who may otherwise not be exposed to the information. Innovative strategies for the provision of reproductive health information appears necessary to reach the variety of age groups and populations of women who are of childbearing age, including WLHIV.

Results illustrate differing paths of reproductive decision-making among WLHIV, reflecting their unique circumstances, and may inform preconception counselling strategies. Future research could potentially target younger women by using motivational interviewing (MI), a tailored strategy designed to move patients towards decision-making and action [38] that has been used to facilitate pregnancy decision-making [39] and promote health behaviour [38–40]. Younger women may be more receptive to MI, which supports patient autonomy and explores ambivalent attitudes and behaviour. In contrast, older or more educated women in this sample were less likely to be influenced by partners than by providers, and may be more receptive to traditional health information provision strategies.

Given its small sample size, results from this study should be used to stimulate further research and not be generalized to recommendations for program or provider practices. The small sample size also limited statistical analysis, since more sophisticated strategies to uncover different “types” of decision makers, such as cluster analysis or latent class analysis, generally require large sample sizes. The analytic strategy utilized only allows comparison of one attribute at a time and required many tests, which may have resulted in false discoveries. Although no participants indicated that the questionnaires were confusing or difficult [41], some may have found the questions repetitive or attempted to move through this theoretical decision-making exercise quickly. It may have also been difficult for women with an HIV-infected partner to imagine having a child with an uninfected partner, and due to the limited sample size, it was not possible to exclude women who did not want a child or had an HIV-infected partner. Finally, this study relied on participant self-reported viral load data, which may be inaccurate.

## Conclusion

This study of WLHIV living in an urban setting identified strong desires to conceive as well as a need to educate WLHIV about safer conception methods. Studies are needed to assess the current implementation of the preconception counselling protocol, to identify which of the many providers seen by WLHIV may be exploring fertility desires, and to explore the use of patient educators and/or nursing staff to more comprehensively provide preconception counselling to WLHIV of reproductive age [42–43]. Strategies assessing combinations of components of realistic scenarios, such as those used in this study, appeared to provide a clearer insight into how and what women might do when faced with real-world scenarios involving trade-offs between the competing elements surrounding pregnancy planning. Research evaluating individual and unilateral aspects of reproductive decision-making (e.g., family, partner, and provider) may be overly simplistic, and may fail to accurately represent the evaluation an individual undergoes when considering safe conception. The emphasis that many women may place on their partners’ desires, in contrast with medical recommendations, highlights the importance of involving men in reproductive healthcare [12]. Men matter; their opinions and desires may be the most influential in making the decision to conceive, although providers should remain aware of the potentially diminishing influence of partners over women’s lifespan.
